# Primary Tumors of the Brain and Central Nervous System in Adults and Children in Sub-Saharan Africa: Protocol for a Scoping Review

**DOI:** 10.2196/66978

**Published:** 2025-04-24

**Authors:** Alhasan Ahmed Badeea Al-Fikri, Mesk Alhammadi, Chiedozie Arum, Mahima Kaur, Kayla Del Biondo, Ibrahim Bani, Victor Mudenda, Sten H Vermund

**Affiliations:** 1 College of Medicine Ajman University Ajman United Arab Emirates; 2 Yale School of Public Health Yale University New Haven, CT United States; 3 School of Medicine University of Zambia Lusaka Zambia

**Keywords:** tumor, cancer, brain, central nervous system, Africa, adults, children, scoping review, PRISMA

## Abstract

**Background:**

In Sub-Saharan Africa (SSA), clinical and research investments for oncology screening, diagnosis, and therapy are exceedingly modest, compared to those in higher-income regions. Diseases that are difficult to prevent or treat, such as primary brain and central nervous system (CNS) tumors, are especially challenging in low-resource settings.

**Objective:**

In order to review and synthesize existing evidence to identify research and service gaps, we will conduct a scoping review to assess epidemiological data, clinical series, and health outcomes associated with brain and CNS tumors in SSA.

**Methods:**

This scoping review is guided by the Scoping Review Chapter of the JBI (Joanna Briggs Institute) Manual for Evidence Synthesis. We will search the following databases: Ovid MEDLINE, Embase, Cochrane Library, Scopus, references from salient publications, and the gray literature, the latter focused on the International Agency for Research on Cancer (IARC) and other major global health organizations. We will review titles and abstracts of potentially eligible studies and then full texts by 2 independent reviewers. We will include data from both primary and CNS cancers in persons of all ages. Data will be abstracted independently using piloted data extraction forms, and we will present results according to the PRISMA-ScR (Preferred Reporting Items for Systematic Reviews and Meta-Analyses extension for Scoping Reviews) and PRISMA-P (Preferred Reporting Items for Systematic Review and Meta-Analyses Protocols) guidelines.

**Results:**

A total of 2857 articles were identified through our search strategy. After title and abstract screening, which was completed on February 23, 2025, by 2 independent reviewers, 222 studies met the eligibility criteria, while 2203 were excluded. Full-text screening began on March 3, 2025, and will be followed by data abstraction and analysis from April 15, 2025, until the end of May 2025. The study is expected to be completed by July 2025.

**Conclusions:**

SSA faces substantial challenges in the diagnosis and treatment of CNS tumors due to health care infrastructure limitations, insufficient reporting, and diagnostic supply shortages. The high fatality rates are attributed to underdiagnosis and misdiagnosis as infectious diseases, despite low incidence rates (IRs). The inadequate neurosurgery facilities and pathology resources further complicate the treatment and prognosis. A scoping review will investigate the true burden of underdiagnosis and gaps in outcomes in children and adults in SSA.

**Trial Registration:**

OSF Registries osf.io/57zvc; https://osf.io/57zvc

**International Registered Report Identifier (IRRID):**

PRR1-10.2196/66978

## Introduction

A significant proportion of new cancer cases worldwide are estimated to occur in low- and middle-income countries (LMICs), but the true prevalence is underestimated due to limited diagnostic capacities [1,2]. Sub-Saharan Africa (SSA) faces challenges in providing the necessary oncology and neurology services for the management of central nervous system (CNS) tumors. Reported cases in SSA are far lower than the actual disease burden due to insufficient health infrastructure for accurate diagnosis, treatment, prognosis, and reporting of tumors, with limited access to drugs, surgery, and modern immunotherapies. Poverty, poor public health surveillance infrastructures, and a lack of services in the vast rural landscape further hinder accurate monitoring of cancer outcomes in SSA, contributing to an underestimation of the true disease burden [3]. Late detection of cancers is common, with later reporting and higher reported case fatality rates among adults and children [4]. Furthermore, the SSA medical workforce in oncology, neurology, and neurosurgery is severely undercapacitated, further impeding the effective management of brain and CNS tumors.

Although less common than other neoplasms, CNS tumors rank comparatively high as a cause of death due to their high case fatality rates. The exact etiologies of these tumors remain largely unknown, with various risk factors, such as genetics and environmental exposures (chemical, nutrition, and radiation) proposed, but the evidence linking them to brain tumors remains inconclusive [5-7]. With over 150 distinct types of brain tumors [8], diagnosing them is a challenge. The diagnosis of brain and CNS tumors in LMICs is less frequent than that of high-income countries [9,10], but we cannot be sure that lower incidence, prevalence, or mortality result from limited surveillance, poor diagnostic capacities, deaths from brain/CNS tumors that are not confirmed as such, poor health care access, and/or population structures that require age-standardized adjustments. A reported standardized incidence rate (SIR) for primary CNS tumors in LMIC in 2008 was estimated to be 3.2-3.9 per 100,000 population, while high-income countries had a far higher SIR of 20.1 per 100,000 population; the authors of this CNS cancers report suggested limited infrastructure for tumor management teams (eg, oncologists, neurologists, pathologists, radiologists, and neurosurgeons) to be contributing to underdiagnosis [10].

In 2022, the African continent accounted for 5% of all global brain/CNS tumors prevalence and 6.4% of deaths from these causes [11,12]. Since over 18% of the estimated 2022 global population was in Africa [12], this suggests either a truly lower incidence or conspicuous underreporting. Childhood brain/CNS tumors were more commonly reported in Africa, with 14.2% incidence rates (IRs) of new global brain/CNS tumor cases and 16.9% of deaths reported in 2022 for children aged 0-18 years old [11,12]. An additional source of underreporting of brain and CNS tumors in Africa is the scarcity of well-established tumor registries [9,10].

Based on the Surveillance, Epidemiology, and End Results (SEER) Program from the National Cancer Institute, childhood brain and other nervous system cancers account for 15.9% of all new childhood cancer cases in the United States [13]. This is similar to the 14.2% reported IRs in Africa, pointing to the fact that childhood CNS tumors are a noteworthy source of cancer morbidity and mortality in both LMICs and high-income countries. However, the similarity in incidence also emphasizes that underreporting and hence, underdiagnosis, in Africa, possibly obscure the actual burden of CNS tumors in SSA. This highlights the necessity to further investigate the epidemiological and clinical characteristics of childhood brain and CNS tumors in LMICs, considering the existent gaps in diagnosis and treatment.

Neuro-oncologic care in East Africa, with a population of over 440 million people in 2021 relied on only 1 neurosurgeon for every 9 million people, in comparison to 1 per 62,500 people in the United States [1,10], with a 1:144 ratio of neurosurgeons in SSA compared to the United States (population-standardized). This vast neuro-oncologic care gap has been noted for children where only 15%-45% received curative options for brain/CNS tumors in LMICs, in comparison to 80% in high-income countries [1]. In SSA, many primary CNS tumors go undiagnosed, as cases with nonspecific symptoms such as headache, ataxia, vomiting, or seizures are often misdiagnosed for different diseases. Even presentation with a visible cranial mass may not result in a pathology-confirmed diagnosis, particularly in rural areas, as only 35% of the developing countries in Africa have pathology resources in their institutions [14]. Despite efforts to diagnose primary CNS tumors in SSA based on global guidelines, challenges persist due to the low per capita total health expenditure in African countries (US $135 in 2010), in comparison to US $3150 in high-income countries. This discrepancy limits the holistic approach to tumor diagnosis, contributing to underreporting and expected high fatality rates [15].

Given the heterogeneity of existing literature on primary CNS tumors in SSA across both pediatric and adult populations, a scoping review is the most suitable method to map the current evidence and evaluate the knowledge state. Our scoping review will investigate a variety of epidemiological and clinical variables, unlike systematic reviews, which address narrow research questions. This will help pinpoint underexplored topics, present data across different age groups, and compare results across different SSA regions, yielding insights that may be missed in narrower reviews.

There is a noteworthy gap in studies that comprehensively explore both epidemiological and clinical data for children and adults with primary CNS tumors in SSA. Existing scoping and systematic reviews often describe either particular populations or disease aspects [14,16], leaving key interconnections between them unexplored. By investigating data from both groups, as well as focusing on the period from 1999 to 2024 when SSA countries incorporated better usage of imaging modalities like computed tomography scans and MRI (magnetic resonance imaging), and World Health Organization (WHO) primary CNS tumors classification was well updated, our review will shed light on the true burden of underdiagnosis and high rates of fatality in SSA.

The aim of our scoping review is to better understand the frequency and outcomes of primary brain/CNS tumors in SSA across both adult and pediatric populations. This includes data interpretation from population-level surveillance registries and clinical health care settings. By considering factors such as age, sex, diagnostic methods, treatment options, and prognostic outcomes, we aim to provide a comprehensive understanding of the epidemiology and distribution of CNS tumors in SSA in the context of regional and sociodemographic differences. We will also investigate the IRs and prevalence to understand the frequency and the burden of tumors. The clinical course of the brain or CNS tumors will be assessed for information on progression, symptoms, complications, and outcomes. We will assess the impact of early diagnosis and treatment, highlighting the benefits and challenges associated with the timely intervention. The insights from this review can facilitate a holistic approach to effective planning and resource allocation, including the establishment of cancer registries in the region.

## Methods

### Objectives

Our scoping review protocol is designed to provide a comprehensive understanding of the epidemiology and distribution of CNS tumors in SSA, considering regional and sociodemographic variations. Methods for inclusion and analysis of articles will be performed according to the updated JBI (Joanna Briggs Institute) guide to scoping review methodology [17]. The main items in the PRISMA-ScR (Preferred Reporting Items for Systematic Reviews and Meta-Analyses extension for Scoping Reviews) and PRISMA-P (Preferred Reporting Items for Systematic Review and Meta-Analyses Protocols) [18,19] will guide the reporting of the protocol process, methods, and findings. The protocol is registered with the Open Science Framework since March 21, 2024, and will be updated.

#### Research Questions

After a preliminary literature review and consultation with experts, we finalized the specific objectives of this scoping review as follows:

1. What is the state of knowledge about brain/CNS tumors in adult and child patients in SSA?
2. What are the best available estimates of SIRs and prevalence of primary CNS tumors, based on SSA population-based surveillance and registries?
3. What are the characterizations of clinical presentations, diagnoses, and outcomes of primary brain/CNS tumors, based on case series from SSA health care settings?

#### Identifying Relevant Studies—Study Selection and Eligibility Criteria

A few studies have covered similar ground, but we seek to update and expand on the existing research conducted on adults and children in SSA [14,16,20,21]. We will use a comprehensive search strategy without age or language restrictions, searching over a 25+ year period (1999 – 2024). Studies based on secondary/metastatic brain tumors (based on the 2021 WHO classification of Brain/CNS tumors), endocrinological tumors, peripheral nervous system tumors, and metastatic tumors are excluded, such that we only review primary brain/CNS neural tumors in persons of all ages. SSA populations are included from the 48 nations considered to be SSA by the World Bank (Textbox 1) [22]. (There are 46 countries considered by the United Nations Development Programme as SSA and 47 countries are in the Africa Region of the WHO).

**Nations in Sub-Saharan Africa as per the World Bank classification.**

Angola; Benin; Botswana; Burkina Faso; Burundi; Cabo Verde; Cameroon; Central African Republic; Chad; Comoros;

Democratic Republic of the Congo; Republic of the Congo; Côte d'Ivoire; Equatorial Guinea; Eritrea; Eswatini; Ethiopia;

Gabon; Gambia; Ghana; Guinea; Guinea-Bissau; Kenya; Lesotho; Liberia; Madagascar; Malawi; Mali; Mauritania; Mauritius;

Mozambique; Namibia; Niger; Nigeria; Rwanda; São Tome and Principe; Senegal; Seychelles; Sierra Leone; Somalia;

South Africa; South Sudan; Sudan; Tanzania; Togo; Uganda; Zambia; Zimbabwe

*Does not include six nations deemed by the World Bank to be in North Africa: Algeria, Djibouti, Egypt, Libya, Morocco, and Tunisia

Data sources include patient series data and population-level data from a 1999-2024 data collection period. Studies published before 1999 are excluded due to limited SSA data availability from that earlier period, the relative local scarcity of diagnostic tools like MRI, and the unavailability of most treatment outcomes in the 20^th^ Century. The inclusion of a given article depends on data contributing to one of the specific objectives of this scoping review, such as clinical presentation, outcome, diagnosis, treatment, prevalence, and/or incidence. Data are eligible from case series, cross-sectional studies, tumor registries, and studies that provide epidemiologic, clinical, and/or outcomes data, Articles that are limited to case studies, systematic reviews, or meta-analyses are excluded in screening but may be relevant to our discussion (as when we compare our findings with previous reviews).

#### Search Strategy

The review team includes a public health/medical librarian (KDB) for assistance with devising a comprehensive search strategy. The first step of this process was an initial limited search in PubMed conducted by a clinical epidemiologist (SHV). From this initial search, the review team identified relevant validation articles and supplied these to the librarian. By reviewing these papers’ title/abstract keywords and index terms (Medical Subject Headings [MeSH]), and by referring to the WHO Classification of Brain Tumors, the librarian worked with the team to devise an in-depth search strategy for MEDLINE (Ovid platform) and other data sources.

As the librarian developed the main database search, the research team met frequently to review the search log and address specific questions on nosology to make final decisions on terms that should or should not be used (for example, the decision to exclude chondro-osseous tumors, even though this is listed in the WHO classification, but to include “chondrosarcoma”).

This search strategy was peer-reviewed according to the Peer Review of Electronic Search Strategies (PRESS) guidance checklist by the Canadian Agency for Drugs and Technology in Health (CADTH) before it was translated to run in the other databases searched for this in-depth scoping review. The other databases that the team used to search for literature are Embase (Ovid platform), Global Health (Ovid platform), Web of Science (Clarivate Analytics PLC), Africa-Wide Information (EBSCO Information Services), and Africa Index Medicus (WHO). A search across all databases was executed in September 2024 (Multimedia Appendix 1). These databases were chosen because of the breadth of medical literature indexed in all the platforms, the availability of literature pertaining to a wide range of countries (especially SSA nations) in the case of Global Health, Africa-Wide Information, and Africa Index Medicus, and the citation network features and gray literature available in Web of Science. To uncover additional gray literature, the authors will search websites (eg, IARC [International Agency for Research on Cancer], WHO, professional organizations) for policy documents on brain/CNS tumor care, best practices, and additional data. As recommended by the JBI Manual for Evidence Synthesi*s* [23], a 3-step search strategy will be used, including a search of reference lists of the included studies for additional sources.

#### Selection of Sources/Screening

The search results obtained from the medical librarian will be imported into Covidence (Cochrane Reviews). After removing duplicate articles, we will proceed with a two-stage screening process, which includes evaluating titles/abstracts and full texts against our predefined eligibility criteria. Each stage of screening will be conducted independently by two reviewers from our screening team (AAA-F, MA, CA, and MK). Before the initial screening of titles and abstracts, we will calibrate our citation screening form through pilot testing. This calibration process will involve three reviewers independently assessing a random sample of 25 citations obtained from the literature search. The team will then convene to discuss any discrepancies with the team’s senior members (IB, VM, or SHV) and refine the eligibility criteria and screening form. Actual screening of titles and abstracts will commence once we achieve at least 75% agreement among the reviewers.

For full-text screening, all articles marked as “include” by two screeners during the title/abstract screening phase will undergo an independent assessment by a third reviewer (VM or SHV) for disparities in judgments. If we are unable to access the full text of any article through the Yale University database or open-access sources, we will use other libraries and/or initiate contact with the first author via email to request a PDF copy of the article, with a goal of securing all studies deemed eligible for our scoping review.

Throughout the review process, we plan to maintain regular communication and collaboration among the screening team members. We will schedule weekly meetings to discuss any observations or insights related to the review process. If necessary, adjustments to the inclusion criteria will be documented and implemented based on these discussions. In addition, we will diligently document reasons for excluding articles during the full-text review phase in Covidence, and we will adhere to the PRISMA-ScR guidelines to create a flow diagram that visualizes the selection process and documents the exclusion reasons.

#### Data Charting and Synthesis of Results

A data extraction form using Covidence software and Microsoft Excel file in addition to the PAGER (Patterns, Advances, Gaps, Evidence for practice and Research recommendations) framework [24] to categorize the data will be used to provide a descriptive outline of results that align with our scoping review objectives. Before implementation, the data extraction form will be pilot-tested using the first 25 studies to ensure that all the information was captured accurately. Any deviations or additions to our data extraction table (Multimedia Appendix 2) will be reported in the final scoping review.

The team will extract the following data for each included study:

- Study characteristics, including year of publication, study type, the period of the study, aims of the study, location of patients or participants, and the number of patients with brain or CNS tumors.
- Patient and tumor characteristics including the origin of data (coming from a registry or a health care setting), sex, age or age range, and the types of any diagnosed brain/CNS tumors. Insofar as possible, we will classify tumors as supratentorial and infratentorial and will provide more detailed tumor anatomical distribution information when available.
- Clinical presentation including diagnoses, therapy highlights, outcome highlights per case, age, and sex, will also be identified. In addition, IRs, SIRs, prevalence, survival rates, mortality rates, and case fatality rates (CFR) will all be identified if available from a specific source.

The 2 reviewers will divide the source load and independently extract data from the included articles. Subsequently, each author will review the extracted data of the other [19]. Discrepancies in extracted data will be discussed between reviewers until consensus is reached or by arbitration of a third reviewer (VM or SHV), as required to ensure the dignity and consistency of the scoping review and accurate data collection. To ensure inter-rater reliability, a 20% sample of included articles independently reviewed will then be compared by the 3 members of the research team.

#### Data Analysis and Presentation

For data analysis and presentation of the results, we will follow the recommendations outlined in the JBI Manual for Evidence Synthesis [23]. In addition, we will use the PAGER framework [24] for scoping reviews to identify recurrent themes and patterns through thematic analysis. This approach will facilitate the identification of key themes and patterns, with frequencies used to summarize the extracted qualitative information. Using SAS software (SAS Institute), quantitative data will be analyzed and presented in tables and graphs. After data extraction, which will be conducted using either a standardized Microsoft Excel file or Covidence (to be determined), the data will be imported into SAS for cleaning and analysis. The qualitative data will undergo thematic analysis to identify recurrent themes and gaps in the literature. The PAGER framework will guide this analysis, categorizing the data into five dimensions: Patterns, Advances, Gaps, Evidence for practice, and Research recommendations that align with our objectives [24].

We will apply thematic analysis to identify key patterns across qualitative data using the qualitative software NVivo (Lumivero), organizing the information by country, tumor type, and context. Thematic analysis will be used alongside narrative synthesis to integrate and interpret the findings. Figures related to the qualitative data will address the following points:

- Treatment modalities
- Diagnostic method used
- Location of tumor: infratentorial/supratentorial—pie chart for different tumor types
- Type of health care facility

Quantitative results will be summarized to highlight frequencies and describe notable patterns and findings relevant to our objectives. The results will be presented in tables and graphs to provide an overview of the key findings. Descriptive statistics will be performed for the numerical data (mean, median, range, and frequency distribution), and demographic will be based on geography, age, gender, and ethnicity.

- Summaries of epidemiological data
- Number of patients to doctors, facilities, and frequency diagnostic tools
- Mortality
- Number of studies and type
- Number of countries included in the paper after screening
- Distribution of brain tumors across years/different regions in SSA / ages

#### Data Visualization

We will summarize IRs, SIRs, prevalence, survival rates, mortality rates, and CFRs using tables. We will also use bar charts to illustrate tumor types, sex, and age distribution for children and adults across different regions. In addition, we will generate heat maps to highlight regional variations in tumor epidemiology.

#### Ethical Considerations

Our literature review was determined exempt from ethical review by the Yale University institutional review board as it does not involve human subjects; all data reviewed are from published sources. We will disseminate our findings through publication, presentations, and targeted sharing with stakeholders. These include oncology-interested community-based organizations and nongovernmental organizations, medical and cancer centers, Ministries of Health, interested professional organizations including oncologists, neurosurgeons, neurologists, and international organizations.

## Results

Initially, we identified 2857 articles relevant to our search strategy. After title and abstract screening, which was completed on February 23, 2025, by 2 independent reviewers using Covidence, we retrieved 222 studies that met our eligibility criteria. A total of 2203 papers were excluded for not meeting the criteria. Full-text screening will commence on March 3, 2025, and is expected to be completed by April 15, 2025. Directly after, data abstraction and charting will begin with 2 independent reviewers to extract the relevant data and key findings, and discrepancies will be resolved by a third reviewer. Data analysis will follow on May 15, 2025, and we aim to complete the final paper by September 2025.

## Discussion

### Principal Findings

Our scoping review focuses on mapping the evidence on CNS tumors in SSA, comprehensively, across pediatric and adult populations. We anticipate to find noteworthy underreporting of CNS tumors in the SSA region attributed to health care infrastructure limitations, diagnostic constraints, and inadequate cancer registries. Data underreporting likely results in an underestimation of the actual burden of CNS tumors in SSA. We additionally expect the review to reveal a high degree of heterogeneity in the available evidence, with variant tumor types, incidence and prevalence rates, and regional outcomes. This variation is expected to mirror differences in health care accessibility, medical workforce strength, and diagnostic criteria availability, which can identify possible areas that necessitate interventions.

Our review will summarize and present data from a broad range of studies to overcome any limitations or fragmentation of the existing literature [14,16]. Comparing our findings to the current data will suggest how SSA countries can adapt these local contexts to best practices. Our targeted populations and the review span of the last 25 years are essential for age-appropriate strategies for diagnosis and treatment, and the assessment of diagnostic improvements in the detection and management of CNS tumors in SSA. Further, this review can provide beneficial insights for health care providers and policymakers, as we assess regional differences in health care infrastructure and access to neuro-oncological care. Nevertheless, there are expected limitations, such as challenges in producing findings from studies with variations in sample size, methodology, and quality, given the heterogeneous studies. The insufficiency of robust cancer registries in many SSA countries, misdiagnosis, and underdiagnosis of CNS tumors, present a challenge in identifying the true incidence and prevalence rates of CNS tumors in SSA.

Understanding the factors that lead to a high incidence of these tumors, and longitudinal studies to examine patient survival and quality of life, should be an area of focus for future studies to improve neuro-oncological care in SSA. Upon the completion of the review, we will seek the dissemination of the findings broadly to ensure that researchers, health care providers, public health organizers, and policymakers have a reach of our review. The aim is to have JMIR as our primary method of dissemination, where our findings can be distributed regionally and globally. We also intend to present the results at international conferences that focus on global health, neuro-oncology, and oncology, which helps in the engagement with many stakeholders.

### Patient and Public Involvement

No patients or members of the public were involved in the review protocol. However, many decades of clinical experience and epidemiological study of tumors in Africa are represented by senior members of the research team (CA, IB, VM, SHV) [25-45].

### Dissemination

Our scoping review aims to provide valuable insights to various stakeholders involved in cancer care and management in Africa. This includes Ministries of Health, international agencies, cancer centers, patient advocates and policymakers, and health care providers and managers including internists, pediatricians, nurses, neurosurgeons, neurologists, hospital directors, and others caring for oncology patients. By conducting this review, we will address key questions to help set priorities for diagnostic, therapeutic, and research action, clarify brain/CNS cancer trends, and better characterize populations most affected. We hope that the timing is favorable now that HIV and AIDS investments have improved chronic care capacities in many SSA nations [46].

### Conclusion

SSA faces a unique set of challenges, including a high burden of infectious diseases and limited health care infrastructure, which increases vulnerability to various health conditions. The lower reported incidence of CNS tumors in this region is likely influenced by underdiagnosis, lack of advanced diagnostic tools, and the absence of comprehensive cancer registries [47]. However, emerging evidence suggests that CNS tumors may be relatively common in this region compared to high-income countries, with certain infections such as Epstein-Barr virus and HIV playing a significant role in their etiology. These infections may complicate the diagnosis and management of CNS tumors, emphasizing the critical need for enhanced diagnostic capacity, targeted research, and effective policymaking to improve health care systems and address this multifaceted challenge.

## Appendix

Multimedia Appendix 1 Search strategy.

Multimedia Appendix 2 Data extraction form for each eligible study reviewed.

## Abbreviations

CADTH: Canadian Agency for Drugs and Technology in Health
CFR: case fatality rate
CNS: central nervous system
IARC: International Agency for Research on Cancer
IR: incidence rate
JBI: Joanna Briggs Institute
LMIC: low- and middle-income country
MeSH: Medical Subject Headings
MRI: magnetic resonance imaging
PAGER: Patterns, Advances, Gaps, Evidence for practice and Research recommendations
PRESS: Peer Review of Electronic Search Strategies
PRISMA-P: Preferred Reporting Items for Systematic Review and Meta-Analyses Protocols
PRISMA-ScR: Preferred Reporting Items for Systematic Reviews and Meta-Analyses extension for Scoping Reviews
SEER: Surveillance, Epidemiology, and End Results
SIR: standardized incidence rate
SSA: Sub-Saharan Africa
WHO: World Health Organization

## Data Availability

The data supporting the findings of this scoping review will be open and accessible to everyone in an appendix of the final manuscript. Extracted data will be provided as supplementary materials accompanying this paper. For more specific information, please contact the corresponding authors.
