# Supplementary material for: Primary Tumors of the Brain and Central Nervous System in Adults and Children in Sub-Saharan Africa: Protocol for a Scoping Review
Source: JMIR Res Protoc. 2025 Apr 24;14:e66978. doi: 10.2196/66978 (PMC12062764; doi:10.2196/66978)
Supplement: Multimedia Appendix 1 [file resprot_v14i1e66978_app1.docx]

Ovid MEDLINE(R) ALL <1999 to September 23, 2024>

1 exp Central Nervous System Neoplasms/

2 exp "Neoplasms, Germ Cell and Embryonal"/

3 exp Nerve Sheath Neoplasms/

4 ((brain or central nervous system or cerebell* or cerebr* or choroid plexus or CNS or cranial or embryon* or epidural or dural or dura mater or germ cell* or glial cell* or glioneuronal or hematolymphoid or hypophysis or hypothalam* or infratentorial or intracerebral or intracranial or intraspinal or leptomeningeal* or mening* or neural ectoderm* or neuro ectoderm* or neuroectoderm* or neuroendocrine or neuroepithel* or nerve sheath or neuronal or nerve tissue* or nervous tissue* or paraspinal or pineal or pituitary or pons angle or pontine or posterior cranial fossa or rathke or rhabdoid or sellar or spinal or spine or subtentorial or supratentorial or trophoblastic) adj3 (adenom* or blastom* or cancer* carcinom* or epithelium or neoplasm* or neuroblastom* or hemangiom* or lymphom* or tumor* or tumour* or sarcoma*)).tw,kw.

5 (astroblastoma* or apudoma* or astrocyt* or cerebrom* or chondrosarcom* or chordom* or craniopharyngiom* or encephelophym* or ependym* or epithelioma medullae* or esthesioneuroepitheliom* or gangliocytom* or gangliogliom* or ganglioneurom* or germinom* or glioblastom* or glioma* or gliosarcom* or glioses or gliosis or hemangioblastom* or liponeurocytom* or medulloblastom* or medulloepithelioma* or medullomyoblastoma* or meningeom* or meningiom* or meningotheliom* or neurilemmom* or neurocytom* or neuroepithelioma* or neurofibrom* or oligodendro* or paragangliom* or pinealom* or schwannom* or spongioblastoma* or teratom*).tw,kw.

6 exp "Africa South of the Sahara"/

7 (south of the sahara or subsaharan africa or angola or benin or botswana or burkina faso or cabo verde or cape verde or cameroon or central african republic or chad or congo or cote d'ivoire or ivory coast or djibouti or equatorial guinea or eritrea or eswatini or ethiopia or gabon or gambia or ghana or guinea or guinea-bissau or kenya or lesotho or liberia or malawi or mali or mauritania or mozambique or namibia or niger or nigeria or rwanda or sao tome or principe or senegal or sierra leone or somalia or south africa or sudan or tanzania or togo or uganda or zambia or zimbabwe or burundi or comoros or madagascar or sahel or somaliland or zahir).tw,kw.

8 (1 or 2 or 3 or 4 or 5) and (6 or 7)

9 8 not ((exp animal/ or nonhuman/) not exp human/)

10 limit 9 to yr="1999 -Current"
