# Supplementary material for: Primary Tumors of the Brain and Central Nervous System in Adults and Children in Sub-Saharan Africa: Protocol for a Scoping Review
Source: JMIR Res Protoc. 2025 Apr 24;14:e66978. doi: 10.2196/66978 (PMC12062764; doi:10.2196/66978)
Supplement: Multimedia Appendix 2 [file resprot_v14i1e66978_app2.docx]

**Table S2.**

| First Author, publication year, Covidence #, PMID #Study characteristics | Kalubula M, 2021, #10, 35233276 |
| --- | --- |
| Type of study |  |
| Study period/Location |  |
| # of tumor patients |  |
| # of CNS tumor patients |  |
| Patient Origin (e.g., clinic, hospital) |  |
| Age Range, mean, S.D., median |  |
| Sex (male or female or unknown) |  |
| Type of Brain/CNS tumor |  |
| Supratentorial/Infratentorial tumor (or more detail) |  |
| Risk factors, e.g., socioeconomic |  |
| Clinical presentation/ Diagnosis |  |
| Therapy and outcome highlights |  |
| Standardized Incidence Rates (SIRs) |  |
| Prevalence |  |
| Survival rates/ Mortality rates |  |
| Case fatality rate (CFR) |  |

Table 2: Data Extraction Form for each eligible study reviewed
